# Supplementary material for: Opioid-blunted cortisol response to stress is associated with increased negative mood and wanting of social reward
Source: Neuropsychopharmacology. 2022 Feb 9;47(10):1798–807. doi: 10.1038/s41386-022-01283-8 (PMC9372154; doi:10.1038/s41386-022-01283-8)
Supplement: Supplementary file 1 — Supplementary Material [file 41386_2022_1283_MOESM1_ESM.pdf]

Supplementary Material

Opioid-blunted cortisol response to stress is associated with increased negative mood and wanting of social reward

C. Massaccesi, M. Willeit, B. B. Quednow, U. M. Nater, C. Lamm, D. Müller, G. Silani

Index

1. *Complete list of exclusion criteria and requirements*
2. *Serum levels of morphine and its metabolites*
3. *Effects of COVID-19 pandemic*
    a. Safety measures
    b. Additional analyses to assess the effects of the COVID-19 pandemic and employed safety measures on responses to social rewards
4. *Social reward stimuli*
5. *Facial electromyography (EMG)*
6. *Physiological and subjective measures of stress*
7. *Drug effects on cognitive functions*
8. *Drug side-effects*
9. *Tables with means and SDs of the stress and reward measures*
10. *Plot of EMG results*
11. *References*

1. Complete list of exclusion criteria and requirements

All participants reported to be right-handed, to smoke less than ten cigarettes per week, to have no history of current or former drug abuse, to have a BMI between 17 and 35, and to be free of psychiatric or neurological disorders. Other exclusion criteria were: single or repeated use of any strong opioids in the last two years, use of hormonal contraceptives, regular intake of medications, current pregnancy or breastfeeding, suffering from impaired respiratory functions, respiratory weakness or lung disease, injury/disease of the arms (making it impossible to squeeze with the right hand and to be caressed on the left forearm). Participants were instructed to refrain from eating, brushing their teeth and consuming caffeinated beverages, juices, and chewing gum in the two hours preceding the test, as well as from smoking, doing physical activity and intaking alcohol and medications in the 24 hours preceding the test.

2. Serum levels of morphine and its metabolites

A blood sample was drawn at the end of the session (~180 min after drug administration). Analyses were performed at the Institute of Clinical Chemistry, University Hospital Zurich, using liquid chromatography coupled to mass spectrometry (LC-MS) to identify serum levels of morphine and its two major metabolites: morphine-3-glucuronide (M3G) and morphine-6-glucuronide (M6G). Blood samples from 3 participants could not be obtained and 10 samples were lost because of storage problems. Results from the available samples confirmed drug uptake, as shown in *Table S1*.

Table S1. Serum levels (nmol/l) of morphine and its major metabolites at the end of the experimental session (~180 min after drug administration). M3G & M6G = morphine-3/6-glucronide.

	**M**	**SD**
Morphine	12.63	6.82
M3G	394.79	179.46
M6G	84.63	44.32

3. Effects of COVID-19 pandemic

Half of the sample was collected after the COVID-19 pandemic outbreak. The implemented safety measures, as well as the statistical analyses conducted to assess the possible effects of the pandemic on the study dependent variables are described below.

a. Safety measures

After the COVID-19 pandemic outbreak the following safety measures were implemented:

- All experimenters wore face masks. The evaluating panel of the TSST wore a special face mask with a clear plastic insert on the mouth region in order to allow the participant to see the "absence of facial feedback" (crucial for stress induction) during the stress paradigm.
- Participants were provided with clear mouth visors resting on the chin in order to minimize the contact with the face and avoid disturbances during facial electromyography (EMG).

In order to assess participants reaction towards these safety measures, they were asked to answer the following questions at the end of the experimental session:

1) "How well/comfortable did you feel during the study?" rated on a VAS ranging from 1 (not all) to 101 (very much).
2) "How high do you rate the risk of infection during the study?" rated on a VAS ranging from 1 (very high) to 101 (very low).
3) "Were you afraid of being infected with COVID-19 during the study?", Yes/No
4) "How comfortable did you feel with the research team wearing a mask during the study?" rated on a VAS ranging from 1 (not all) to 101 (very much).

As shown in Figure S1, participants did not report to feel threatened by COVID-19 infection during the study, and to feel comfortable during the session.

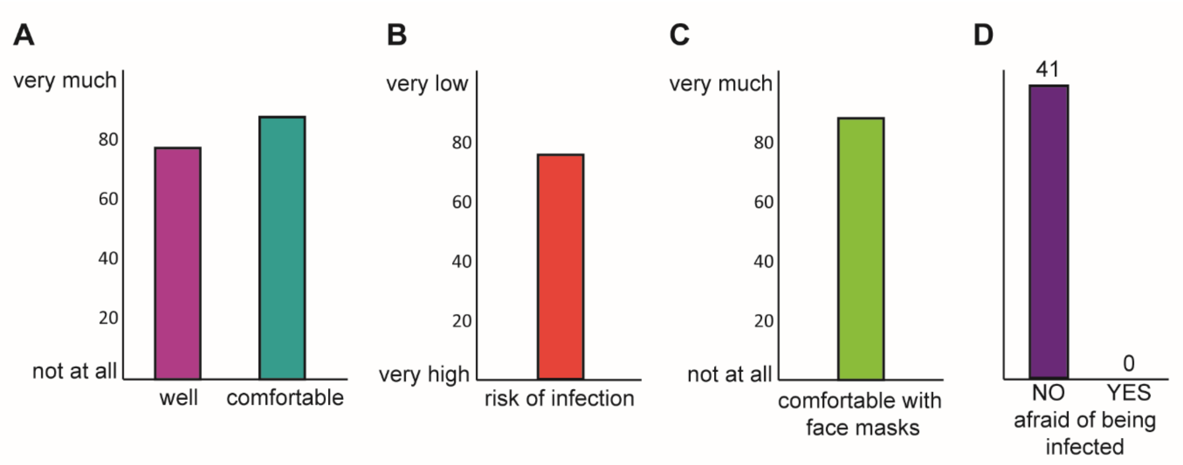

Figure S1. Participants' attitudes toward COVID-19 pandemic and safety measures during the study. (A) Ratings of wellness and comfort. (B) Perceived risk of COVID-19 infection during the study. (C) Ratings of comfort related to use of face masks during the study. (D) Perceived fear of having been infected with COVID-19 during the study.

b. Additional analyses to assess the effects of the COVID-19 pandemic and employed safety measures on responses to social rewards

We tested for possible effects of the pandemic and related implemented safety measures on the subjective stress response (positive and negative mood, POMS subscales), as well as on the ratings of wanting and liking, on the force exerted to obtain the social rewards, and on facial EMG data, by adding the covariate "COVID-19" (2 levels: pre-covid19, covid19) to the statistical models. No changes in the pattern of results were observed.

4. Social reward stimuli

The suitability of the stroking speeds (6, 21 and 27 cm/s) has been confirmed in three previous independent studies from our group [1–3]. Caresses were delivered over a previously-marked (from the wrist towards the elbow) 9 cm area on the participant's left forearm by a female experimenter, moving her index and middle fingers back and forth in the marked area (Figure 1B). Touch delivery was guided by auditory rhythms, which matched the frequency of the stimulation, over headphones. The experimenter administering the touch was seated on the other side of a curtain, used to limit the participant's field of view to the monitor (Figure 1B). All experimenters were presented as trained masseurs, wore standardized clothes (white t-shirt and beige trousers) to minimize differences in their appearance, and underwent extensive training on the tactile stimulation delivery.

5. Facial electromyography (EMG)

Facial EMG was recorded throughout the Social Reward task, using a g.USBamp amplifier (g.tec Medical Engineering GmbH) and the software Matlab (MathWorks, Inc). Participant's face areas were prepared using alcohol, water and an abrasive paste. Reusable Ag/AgCl electrodes were then attached bipolarly according to guidelines [4] on the left corrugator supercilii and zygomaticus major muscles. A ground electrode was attached to the participant's forehead and a reference electrode on the left mastoid. The EMG data were sampled at 1200 Hz with impedances below 20 kΩ. Data preprocessing included filtering with a 20 to 400 Hz bandpass filter and a 50Hz notch filter, rectification and smoothing with a 40 Hz low-pass filter.

Each trial was divided in 4 epochs: Anticipation Pre-Effort (announcement of best attainable reward, 3 s), Anticipation Post-Effort (announcement of attained reward, 2 s), Delivery (touch administration, 6 s), and Relax (relax phase after reward delivery, 5 s) (Figure 1C). EMG was first averaged over 1 s time-windows and then over the epoch total duration. For each trial, values in the four epochs were expressed as percentage of the average amplitude during the fixation cross at the beginning of that trial (baseline, 2 s). Outliers in baseline values (defined as values more than 3 SDs away from the subjects' average baseline) were substituted with the average amplitude of the baseline preceding and following that trial. The extracted epochs were visually inspected to identify signal artifacts which were then removed (33 epochs for corrugator and 51 epochs for zygomaticus across 5 participants). Because of data skewness and to reduce the effect of non-experimental movements, for each subject, epochs over the subject's mean ± 3 SD were removed (average removed epochs per subject: corrugator: $M = 2.4$, $SD = 1.2$; zygomaticus: $M = 3.1$, $SD = 1.1$), and the remaining data were transformed using natural logarithmic transformation.

6. Physiological and subjective measures of stress

Free cortisol concentration in saliva was determined by using commercial luminescence immunosorbent assay (LUM; IBL, Hamburg, Germany). Salivary alpha-amylase activity was measured using a kinetic colorimetric test and reagents obtained from DiaSys Diagnostic Systems (Holzheim, Germany). For heart rate, salivary cortisol and alpha-amylase analyses, outliers were defined as subjects with a baseline value (T1) 3 SDs over the mean baseline of the sample. This procedure led to the exclusion of two participants for cortisol (1 MORPH, 1 PLB) and of one participant for alpha-amylase (MORPH).

The in-house mood scale consisted of 8 items (happiness, calmness, relaxation, feeling good, stress, tension, anxiety and feeling bad) assessed using VAS ranging from “not at all” (+1) to “very much” (+101). Positive and negative mood items were averaged to constitute the “Positive mood” and “Negative mood” scales used for statistical analyses. The German short version Profile of Mood States (POMS) [5] consists of 4 subscales for current mood: anger, depression, vigor, and fatigue.

7. Drug effects on cognitive functions

Table S2. Mean (SD) scores on the Trial Making Test (TMT) part A and part B, and the Digit Symbol Substitution Test (DSST) across drug groups.

	MORPHINE	PLACEBO	*p* value
TMT A	26.33 (8.02)	26.40 (8.60)	0.97
TMT B	57.25 (33.05)	58.93 (21.94)	0.79
DSST	54.20 (8.99)	54.10 (9.32)	0.96

8. Drug side-effects

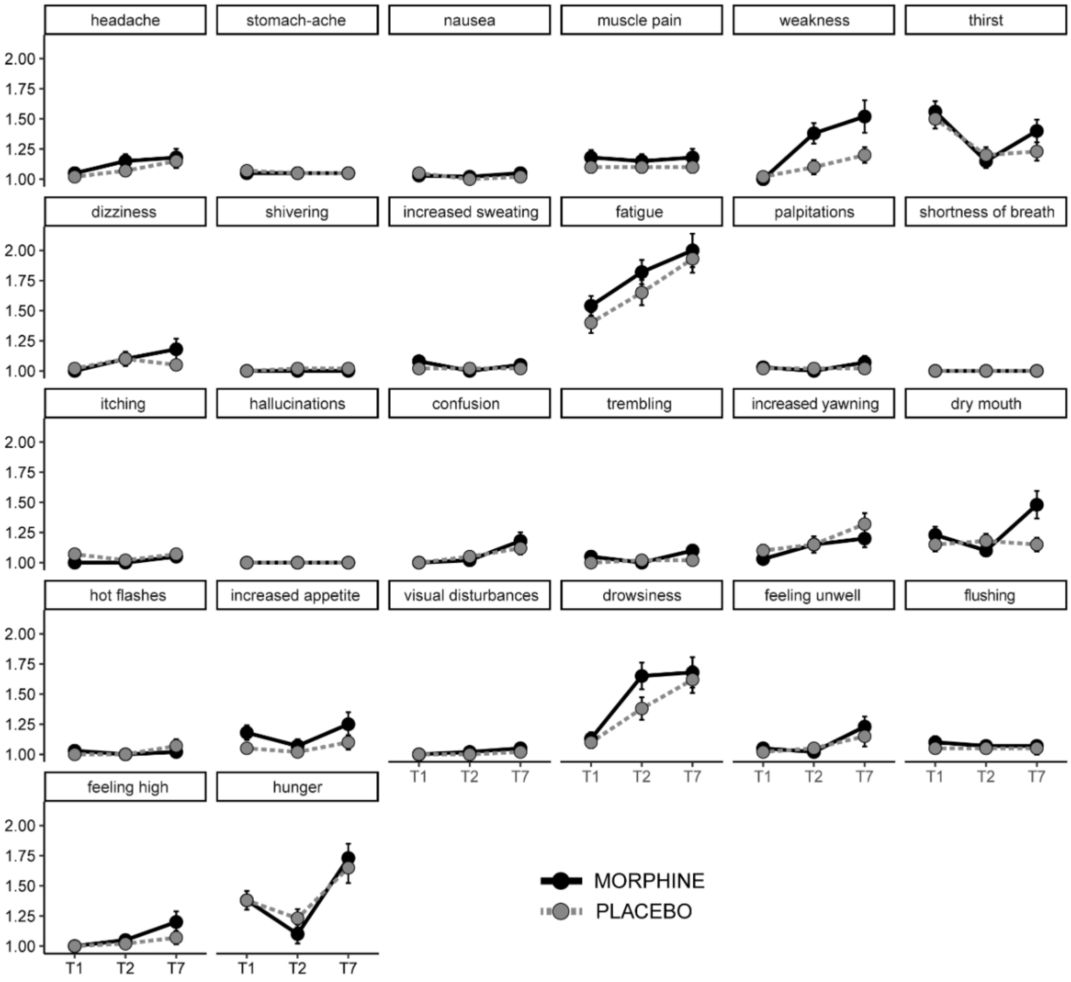

Figure S2. Drug side-effects assessed at baseline (T1), 60 min (T2) and 160 min (T7) after drug administration using a 4-point Likert scale (with the anchors 1 = “not at all” and 4 = “very much”).

9. Tables with means and SDs of the stress and reward measures

Table S3. Means ± SDs of salivary cortisol, salivary alpha-amylase and heart rate.

Cortisol	T1	T2	T3	T5	T6	T7
Morphine	3.1±1.9	1.9±1.0	1.4±0.6	1.3±0.6	1.1±0.5	1.0±0.5
Placebo	3.5±2.0	2.7±1.8	2.8±3.8	4.0±3.3	4.0±2.4	3.2±1.8
Alpha-amylase	T1	T2	T3	T5	T6	T7
Morphine	69.5±54.1	83.2±52.0	91.0±67.5	161.3±145.5	127.0±114.8	98.5±76.9
Placebo	56.3±44.0	74.4±53.2	84.5±74.0	159.9±162.5	100.9±80.9	88.7±82.3
Heart rate	Baseline		TSST		Social Reward task	
Morphine	80.2 ± 9.7		97.3 ± 17.7		71.4 ± 9.3	
Placebo	78.4 ± 12.8		92.5 ± 13.1		72.4 ± 10.9	

T1, immediately before drug admin.; T2, 50 min after drug admin.; T3, before TSST (100 min after drug admin.); T5, after TSST (120 min after drug admin.); T6, after the first block of the Social Reward task (140 min after drug admin.); T7, after the second block of the Social Reward task (160 min after drug admin.). Salivary cortisol in nmol/L; Salivary alpha-amylase in U/mL; Heart rate in bpm.

Table S4. Means ± SDs of subjective measures of mood (VAS and POMS).

	T1	T2	T3	T4	T5	T6	T7
Negative mood							
Morphine	12.4±11.8	8.9±11.1	11.3±12.4	34.1±21.9	34.9±27.0	10.6±10.6	7.7±8.3
Placebo	12.4±10.1	9.5±9.7	9.7±9.6	30.6±23.6	25.2±20.1	10.1±10.8	9.7±12.4
Positive mood							
Morphine	71.9±13.6	76.3±15.6	71.4±19.2	43.9±25.2	41.9±26.0	67.1±23.8	71.1±21.6
Placebo	67.0±19.2	69.0±22.4	65.2±21.4	40.1±25.4	44.7±26.7	61.6±24.8	63.1±23.3
Depression POMS							
Morphine	17.1±4.7	16.7±4.6	17.7±6.4	---	26.4±15.0	---	17.4±6.4
Placebo	18.1±5.3	16.9±4.6	16.8±6.4	---	22.8±14.8	---	18.3±10.4
Anger POMS							
Morphine	8.0±2.4	7.9±2.0	8.4±2.7	---	14.5±9.6	---	9.7±4.4
Placebo	8.4±2.2	8.0±1.9	8.4±3.0	---	11.5±5.9	---	9.1±3.6
Fatigue POMS							
Morphine	11.1±4.4	14.0±6.1	17.4±8.4	---	15.2±8.4	---	17.0±8.8
Placebo	12.7±5.3	12.3±6.9	15.2±8.3	---	13.9±7.9	---	15.9±9.6
Vigor POMS							
Morphine	28.0±6.7	22.6±7.2	20.1±7.4	---	21.5±9.7	---	21.0±9.2
Placebo	26.8±6.7	24.4±8.1	21.5±8.6	---	20.9±9.5	---	20.6±9.3

T1, immediately before drug admin.; T2, 50 min after drug admin.; T3, before TSST (100 min after drug admin.); T4, during the TSST preparation phase; T5, after TSST (120 min after drug admin.); T6, after the first block of the Social Reward task (140 min after drug admin.); T7, after the second block of the Social Reward task (160 min after drug admin.). Min-max range: 1-101 for positive and negative mood; 7-49 for Anger, Fatigue and Vigor; 14-98 for Depression. POMS, Profile of Mood States.

Table S5. Means ± SDs of Primary and Secondary Appraisal (PASA) questionnaire and ratings of TSST performance satisfaction.

	MORPHINE	PLACEBO
PASA		
Primary appraisal	15.5 ± 3.1	15.0 ± 3.5
Secondary appraisal	16.1 ± 2.9	16.1 ± 4.4
Performance satisfaction		
Speech	36.3 ± 29.9	40.3 ± 26.3
Math	32.7 ± 26.4	36.1 ± 21.7

Table S6. Means ± SDs of ratings of wanting and liking, and force exerted in the Social Reward task.

	Wanting	Liking	Force
High reward			
Morphine	4.1 ± 3.9	5.0 ± 3.5	76.8 ± 19.2
Placebo	2.0 ± 3.7	2.9 ± 3.3	73.9 ± 17.2
Low reward			
Morphine	0.7 ± 2.9	2.5 ± 2.7	66.9 ± 19.2
Placebo	0.9 ± 2.1	1.6 ± 2.3	70.9 ± 15.7
Very low reward			
Morphine	---	0.6 ± 3.0	---
Placebo	---	0.8 ± 2.6	---

Table S7. Means ± SDs of activity (log transformed) of the Corrugator and Zygomaticus muscles in the Social Reward task.

	log Corrugator	log Zygomaticus
Anticipation Pre-Effort		
Morphine	4.61 ± 0.06	4.59 ± 0.12
Placebo	4.58 ± 0.13	4.57 ± 0.17
Anticipation Post-Effort		
Morphine	4.59 ± 0.24	4.93 ± 0.44
Placebo	4.62 ± 0.27	4.80 ± 0.43
Delivery		
Morphine	4.48 ± 0.18	4.50 ± 0.16
Placebo	4.48 ± 0.16	4.52 ± 0.24
Relax		
Morphine	4.55 ± 0.10	4.52 ± 0.13
Placebo	4.50 ± 0.14	4.58 ± 0.23

10. Plot of EMG results

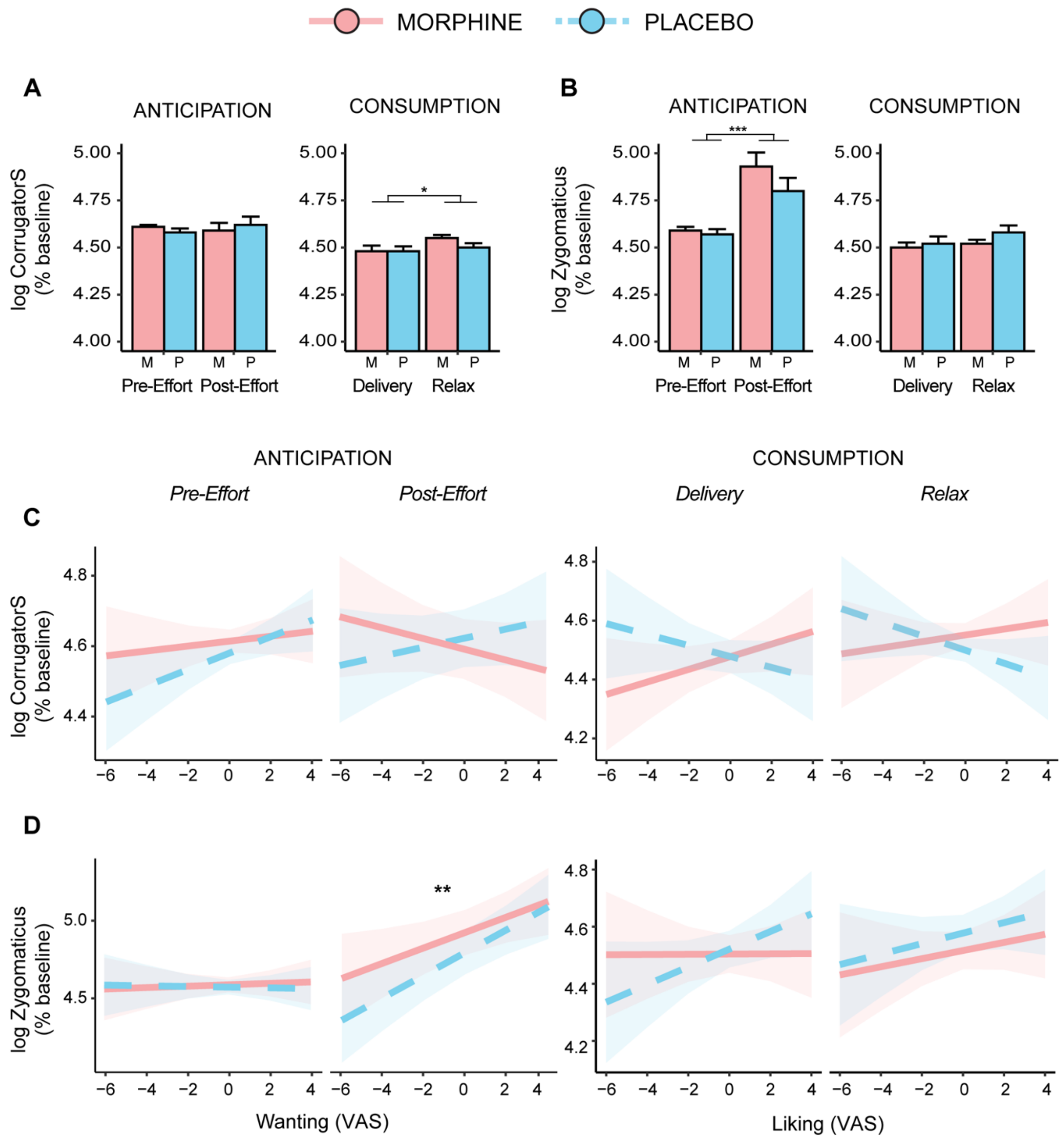

Figure S3. Mean activity (expressed as % of the baseline and log transformed) of the corrugator supercilii (A,C) and zygomaticus major (B,D) muscles by task phase (Anticipation Pre-Effort, Anticipation Post-Effort, Delivery and Relax) and drug (Morphine, Placebo) (A,B), and by task phase, drug and ratings of wanting/liking (C,D). Error bars represent standard error of the mean; ribbons represent 95% confidence interval; asterisks indicate significant differences between conditions (* $p < .05$, ** $p < .01$, *** $p < .001$). M, Morphine; P, Placebo; VAS, Visual Analogue Scale.

11. References

1. Massaccesi C, Korb S, Skoluda N, Nater UM, Silani G. Effects of Appetitive and Aversive Motivational States on Wanting and Liking of Interpersonal Touch. Neuroscience. 2021;464:12–25.
2. Korb S, Massaccesi C, Gartus A, Lundström JN, Rumiati R, Eisenegger C, et al. Facial responses of adult humans during the anticipation and consumption of touch and food rewards. Cognition. 2020;194:104044.
3. Korb S, Götzendorfer SJ, Massaccesi C, Sezen P, Graf I, Willeit M, et al. Dopaminergic and opioidergic regulation during anticipation and consumption of social and nonsocial rewards. ELife. 2020;9:e55797.
4. Fridlund AJ, Cacioppo JT. Guidelines for Human Electromyographic Research. Psychophysiology. 1986;23:567–589.
5. Albani C, Blaser G, Geyer M, Schmutzer G, Brähler E, Bailer H, et al. The German short version of 'Profile of Mood States' (POMS): psychometric evaluation in a representative sample. PPmP - Psychother · Psychosom · Med Psychol. 2005;55:324–330.
